# Transcriptome analysis and metabolic profiling reveal the key role of α-linolenic acid in dormancy regulation of European pear

**DOI:** 10.1093/jxb/ery405

**Published:** 2018-12-26

**Authors:** Gilad Gabay, Adi Faigenboim, Yardena Dahan, Yacov Izhaki, Maxim Itkin, Sergey Malitsky, Yonatan Elkind, Moshe A Flaishman

**Affiliations:** 1 Institute of Plant Sciences, Volcani Research Center, Derech Hamacabim, Rishon Lezion, Israel; 2 The Robert H. Smith Institute of Plant Sciences and Genetics in Agriculture, Faculty of Agriculture, Food and Environment, the Hebrew University of Jerusalem, Rehovot, Israel; 3 Life Science Core Facilities, Weitzman Institute of Science, Rehovot, Israel

**Keywords:** Bud dormancy, European pear, metabolic profiling, QTL, RNA-Seq, transcriptome analysis, vegetative budbreak

## Abstract

Deciduous trees require sufficient chilling during winter dormancy to grow. To decipher the dormancy-regulating mechanism, we carried out RNA sequencing (RNA-Seq) analysis and metabolic profiling of European pear (*Pyrus communis* L.) vegetative buds during the dormancy phases. Samples were collected from two cultivars that differed greatly in their chilling requirements: ‘Spadona’ (SPD), a low chilling requirement cultivar; and Harrow Sweet (HS), a high chilling requirement cultivar. Comparative transcriptome analysis revealed >8500 differentially expressed transcripts; most were related to metabolic pathways. Out of 174 metabolites, 44 displayed differential levels in both cultivars, 38 were significantly changed only in SPD, and 15 only in HS. Phospholipids were mostly accumulated at the beginning of dormancy, sugars between before dormancy and mid-dormancy, and fatty acids, including α-linolenic acid, at dormancy break. Differentially expressed genes underlying previously identified major quantitative trait loci (QTLs) in linkage group 8 included genes related to the α-linolenic acid pathway, 12-oxophytodienoate reductase 2-like, and the *DORMANCY-ASSOCIATED MADS-BOX* (*DAM*) genes, *PcDAM1* and *PcDAM2*, putative orthologs of *PpDAM1* and *PpDAM2*, confirming their role for the first time in European pear. Additional new putative dormancy-related uncharacterized genes and genes related to metabolic pathways are suggested. These results suggest the crucial role of α-linolenic acid and *DAM* genes in pear bud dormancy phase transitions.

## Teaser

New dormancy-associated regulatory genes and metabolites were identified in European pear bud, suggesting the key role of α-linolenic acid and *DAM* genes in dormancy phase transition.

## Introduction

Pear (*Pyrus*) belongs to the Rosaceae family and is of major economic importance in temperate climate regions (Chen *et al.*, 2014; Yamamoto and Terakami, 2016). Although dormancy has a critical effect on fruit growth regulation, the mechanism governing pear bud dormancy is still obscure (Ito *et al.*, 2018; Li *et al.*, 2018). The pear genome and transcriptome data are available and were published in Wu *et al.* (2013).

In perennial plants, dormancy is an evolutionary mechanism developed in temperate regions to adapt to severe cold climate conditions. Rosaceae fruit tree species, including pear, enter into endodormancy in response to decreasing temperatures (Erez and Lavee, 1971; Horvath *et al.*, 2003). Dormancy break occurs when a sufficient number of chilling hours (chilling units=CUs), to which deciduous trees are exposed during endodormancy, have accumulated (Anderson *et al.*, 1986; Heide and Prestrud, 2005). Chilling requirements define the number of CUs needed for the tree to undergo budbreak in the spring, when the climate conditions are suitable for active vegetative growth. Chilling requirements vary widely between cultivars and species (Erez and Lavee, 1971; Wigge, 2013). When chilling requirements are not fulfilled, the vegetative budbreak (VB) date is delayed. Hence, individuals with a high chilling requirement that grow in warm areas where CU accumulation is not sufficient will undergo dormancy break at a later stage; this will affect their vegetative growth, essential for flower and fruit development (Erez and Lavee, 1971; Gabay *et al.*, 2017).

According to climate model predictions, global warming will result in a reduction in CU accumulation during the winter (Campoy *et al.*, 2011). This may lead to severe disorders in deciduous fruit tree growth habits, such as yield reduction and abnormal fruit set (Lang *et al.*, 1987; Wu *et al.*, 2017). Therefore, the genetic mechanism underlying dormancy and the genetic variance associated with dormancy release must be investigated to cope with climate change (Chuine and Beaubien, 2001; Niu *et al.*, 2016).

Genetic determination of chilling requirements is a complex mechanism which is assumed to be governed by multiple genes (Howe *et al.*, 2000). A major advance was achieved in understanding the genetic factors associated with dormancy via the detection of MADS-box genes involved in dormancy (Bielenberg *et al.*, 2008). Six genes termed *DORMANCY-ASSOCIATED MADS-BOX* (*DAM*) were first identified in peach [*Prunus persica* (L.) Batsch]. These genes play a major role in bud set, regulation of vegetative growth, and growth cessation (Jiménez *et al.*, 2010). Gene expression studies in pear have indicated a reduction in the expression level of *PpMADS13-1*, a *Pyrus pyrifolia* (Japanese pear) *DAM* homolog, before dormancy break (Saito *et al.*, 2015). In addition, two other *DAM* genes showed expression levels that were correlated with dormancy phases (*PpMADS13-2* and *PpMADS13-3*) (Saito *et al.*, 2013). To date, three *DAM* genes, *PpDAM1* (previously *PpMADS13-1*), *PpDAM2* (previously *PpMADS13-2*), and *PpDAM3* (previously *PpMADS13-3*), have been identified in pear (*Pyrus* spp.) (Tuan *et al.*, 2017).

Detection of quantitative trait loci (QTLs) can greatly improve accuracy when searching for candidate gene locations. Hence, QTL regions are associated with a trait that is likely to be governed by genes within the QTL intervals (Korstanje and Paigen, 2002). QTLs associated with time of dormancy release have been detected in Rosaceae members, such as apple (van Dyk *et al.*, 2010; Celton *et al.*, 2011; Allard *et al.*, 2016) and peach (Fan *et al.*, 2010). QTLs associated with VB time in European pear were identified in linkage groups (LGs) 5, 8, 9, 13, and 15, representing the only attempt to detect a chilling requirement QTL in *Pyrus* spp. (Gabay *et al.*, 2018). The major QTL was detected in LG8 (*R*^2^=28%). ‘Metabolic pathway’, ‘biosynthesis of secondary metabolites’, and ‘plant hormone signal transduction’ are annotations that were remarkably well represented in cherry dormancy transcriptome analysis according to Kyoto Encyclopedia of Genes and Genomes (KEGG) pathway analysis (Zhu *et al.*, 2015). ‘Metabolite biosynthesis’, ‘ribosome’, ‘starch and sucrose metabolism’, and ‘flavonoid biosynthesis’ pathways were among the most prominent KEGG categories in grape (Khalil-Ur-Rehman *et al.*, 2017*a*). Similar results were obtained in gene expression studies of Chinese pear (Liu *et al.*, 2012; Bai *et al.*, 2013).

Along with differentially expressed genes (DEGs) associated with dormancy phase transitions, numerous metabolites and proteins, such as dehydrins, sugars, fatty acids, and protein kinases, have been shown to be significantly changed in content during the dormancy period (Maruyama *et al.*, 2009; Eremina *et al.*, 2016). Lipid accumulation in the bud meristem is essential for establishing endodormancy, which provides tolerance to cold temperatures. The metabolic processes in bud membranes during dormancy change the lipid composition, leading to optimal conditions for budbreak after exposure to a sufficient number of CUs during the winter, and active bud growth under favorable conditions in the spring (Wang and Faust, 1990). Several studies indicated the specific role of α-linolenic and linoleic acids in dormancy regulation. α-Linolenic acid promotes dormancy break in Japanese pear (Sakamoto *et al.*, 2010). In apple, the increase of linoleic acid was observed when the chilling requirement (CR) is fulfilled (Wang and Faust, 1990). α-Linolenic acid is a precursor in the synthesis of the plant hormone jasmonic acid (JA) (Eremina *et al.*, 2016). JA treatment was reported to promote dormancy break in horse chestnut (Taniguchi *et al.*, 2003). In annuals such as maize and Arabidopsis, JA is an important factor in stamen and pollen maturation (Smith and Zhao, 2016).

Abscisic acid (ABA) is strongly linked to dormancy phase transitions (Ophir *et al.*, 2009). Increasing ABA content in pear was detected during the establishment of bud endodormancy, and decreasing levels were detected toward dormancy break (Li *et al.*, 2018).

The accumulation of raffinose (oligosaccharide) was detected during the establishment of apple dormancy and it was suggested to have a role in protecting the dormant buds against limited availability of free water (Falavigna *et al.*, 2018).

This study provides deep insight into dormancy transitions in pear by looking at the metabolite profiles during dormancy phases combined with transcriptome analysis of bud dormancy in two cultivars that differ greatly in their chilling requirements. In addition, these results were co-analyzed with the expression of genes underlying previously described QTLs associated with timing of VB (Gabay *et al.*, 2018).

## Materials and methods

### Plant material

**Fig. 1.**
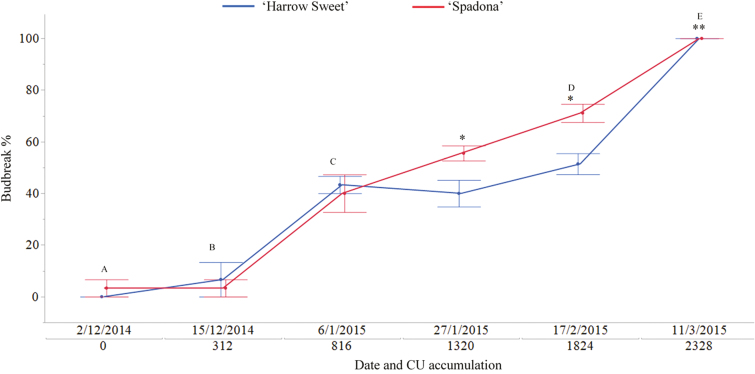
Differences in vegetative bud break percentage between low chilling requirement cv. Spadona and high chilling requirement cv. Harrow Sweet at different stages of tree dormancy. The *x*-axis shows the examination date (dd/mm/yyyy) and chilling unit (CU) accumulation on that date. The *y*-axis shows budbreak percentage after 21 d of growth in the controlled room. Sprout examination time is indicated by dots (red=SPD, blue=HS). Letters indicate sample collection subjected to RNA-Seq and metabolite profiling. *Significant difference between bud break percentages of cultivars. **On this date, the trees were transferred from the chilling controlled room (4 °C) to a natural environment with no CU accumulation. For the samples collected on this date, RNA-Seq and metabolite profiling were performed on the first day of vegetative bud break (HS=19/03/2015, SPD=16/03/2015).

We used two European pear (*Pyrus communis* L.) cultivars that differ greatly in their chilling requirements to evaluate differences and similarities in gene expression: the low chilling requirement (300 CUs) cv. Spadona (SPD) and the high chilling requirement (800 CUs) cv. Harrow Sweet (HS). Five mature (10-year-old) trees of each cultivar, planted in 50 liter pots and grafted on quince rootstock, were placed in a climate-controlled room on 1 December 2014; at that time, no CUs had been accumulated in Bet Dagan, Israel, where the trees had been located prior to the experiment. However, according to the dormancy evaluations, the trees were at a dormant stage (Fig. 1) at that time. Trees were not chemically treated during the experimental period. The room temperature was set to 4 °C, such that 1 h in the controlled room was equivalent to one accumulated CU according to a dynamic model (Erez *et al.*, 1988). Five biological replicates of vegetative buds for RNA sequencing (RNA-Seq; two replicates) and for metabolic profiling (three replicates) were collected randomly from all trees of each cultivar at time 0 (the day the trees were placed in the room), 2 weeks later to evaluate the trees’ first response to low temperatures, and then at 21 d intervals to evaluate the transcriptome and metabolite profiles during representative phases of dormancy. To ensure that only vegetative buds were collected, no apical buds were sampled (i.e. lateral buds were collected). Based on our observations, only the apical buds developed into flowers, due to apical dominance. At the same time of each sample collection, shoots were collected for evaluation of dormancy status. The last sample was collected after the trees had been taken out of the controlled room to natural conditions and first VB was observed, as described in Table 1. The collected samples were immediately frozen in liquid nitrogen and stored at –80 °C until RNA and metabolite extractions.

**Table 1.** Collection dates of vegetative buds for RNA-Seq and metabolite profiling

Collection	Date (dd/mm/yyyy)	Chilling units accumulated^*b*^
Collection 0	01/12/2014	0
Collection 1	15/12/2014	312
Collection 2	06/01/2015	816
Collection 3	27/01/2015	1320
Collection 4	17/02/2015	1824
Collection 5	First budbreak^*b*^	2328^*c*^

^*a*^ CU accumulation according to a dynamic model.

^*b*^ First vegetative budbreak date: HS=19/03/2015, SPD=16/03/2015.

^*c*^ Last day in 4 °C rooms 11 March 2015.

### Evaluation of dormancy status

To examine the differential expression of genes between different dormancy phases, dormancy status was evaluated at each collection time in at least five shoots (length ≥30 cm) with more than five vegetative buds per shoot (30 buds in total) pruned from both cultivars. For bud break induction, the shoots were placed in 500 ml vases containing distilled water and were kept in a controlled-climate room at 24 ± 1.0 °C under a 16 h photoperiod to induce budbreak. Distilled water in the vases was changed every 2–3 d. The dormancy status was determined at each collection time after the evolution of the percentage of the VB in each shoot after 21 d in the controlled room, as described in Ubi *et al.* (2010). Significant differences (*P*<0.05) in percentage VB between collection times within and between cultivar samples were determined with JMP Pro 13 (SAS Institute, Cary, NC, USA) using ANOVA.

### RNA extraction and sequencing

Total RNA was isolated from lateral vegetative buds (100 mg) using the Plant Total RNA Purification kit (Norgen Biotek, Thorold, Canada) following the manufacturer’s instructions. Samples from five collection times were subjected to RNA-Seq according to evaluated dormancy status. In total, 20 samples were used for RNA-Seq (2 cultivars×5 collection times×2 biological replicates) after discarding one collection time (27 January 2015) due to the similarity of VB percentage to the other collection time (Fig. 1). RNA quantity was determined in a NanoDrop 1000 spectrophotometer, and quality was analyzed using the 2200 TapeStation System (Agilent Technologies, Santa Clara, CA, USA) according to the manufacturer’s protocol. The purity of all RNA samples was assessed by 260/280 nm and 260/230 nm absorbance ratios, and they had an RNA integrity number (RIN) of >7 according to TapeStation measurements. Library construction and sequencing were performed by the Genomics Unit at the Grand Israel National Center for Personalized Medicine, Weizmann Institute of Science (Rehovot, Israel). Briefly, the poly(A) fraction (mRNA) was purified from 500 ng of total RNA, followed by fragmentation and generation of double-stranded cDNA. Then end repair, A base addition, adaptor ligation, and PCR amplification steps were carried out. Sequencing libraries were constructed with barcodes to allow the multiplexing of 10 samples in one lane (total of 20 samples in two lanes). An Illumina HiSeq 2500 V4 instrument was used to sequence single end non-stranded 60 bp reads. The number of reads was similar for all samples.

### Filtering and mapping of reads to the reference genome

The collected raw short reads were subjected to a filtering and cleaning procedure. The SortMeRNA tool was used to filter out rRNA (Kopylova *et al.*, 2012). Adaptors were removed using Trimmomatic software, version 0.32 (Bolger *et al.*, 2014). Then, the FASTX Toolkit (http://hannonlab.cshl.edu/fastx_toolkit/index.html, version 0.0.13.2) was used to (i) trim read-end nucleotides with quality scores <30, with the Fastq_quality_trimmer, and (ii) remove reads with <70% of base pairs with a quality score ≤30 using the Fastq quality filter. Transcript quantification (the number of reads per gene) from the RNA-Seq data was performed using the Bowtie2 aligner (Langmead *et al.*, 2009), the *Pyrus* reference genome (Wu *et al.*, 2013), and the expectation–maximization method (RSEM), by estimating maximum likelihood expression levels (Li and Dewey, 2011) via the Perl script align_and_estimate_abundance.pl with the --est_method RSEM from the Trinity protocol (Haas *et al.*, 2013). Correlation between the samples was estimated by a principal component analysis (PCA) graph and was generated using R software based on the expression data (Supplementary Fig. S1 at *JXB* online).

### Differential expression analysis

All successfully mapped transcripts were subjected to differential expression analysis using the DESeq2 R package (Love *et al.*, 2014) between collection times within each cultivar. The expression level was calculated as trimmed mean of M values (TMM)-normalized counts (Robinson and Oshlack, 2010). Transcripts with log2 fold change (Log2FC) values >2 (up-regulated) or < –2 (down-regulated) and with an adjusted *P*-value (*P*_adj_ <0.001) were considered significant. To evaluate differential gene expression that is associated with dormancy status, based on the dormancy status evaluations, the collection times were grouped, corresponding to VB%, into three major stages: ED=entry into dormancy, 10% >budbreak, MD=mid-dormancy, 50% >budbreak, DB=dormancy break, 50% <budbreak. Therefore, DEGs between two collection time points that had no significant differences (e.g. time A versus time B) in VB% were not further analyzed since we cannot correlate those differences of gene expression to the dormancy status. A Venn diagram to visualize the similar and unique numbers of differentially expressed transcripts for each dormancy stage was generated with the Venny tool (http://bioinfogp.cnb.csic.es/tools/venny/; Oliveros, 2007). Transcripts were divided into two categories: up- and down-regulated between the dormancy stages, and plotted with JMP.

### Gene Ontology (GO) annotation and KEGG pathway analysis of DEGs

The *Pyrus* reference transcriptome (Wu *et al.*, 2013) extracted from the NCBI was used as a query for a search of the NCBI non-redundant (nr) protein database using the DIAMOND program (Buchfink *et al.*, 2015). The search results were imported into Blast2GO version 4.0 (Conesa *et al.*, 2005) for GO assignment. GO-based enrichment analysis was carried out to characterize the differential expression of transcripts using the Blast2GO program based on Fisher’s exact test (Upton, 1992) with multiple testing correction of the false discovery rate (FDR) (Benjamini and Hochberg, 1995). The threshold was set as an FDR with corrected *P*-value of <0.05. GO analysis was performed by comparing the GO terms in the test sample with those in a background reference. Three comparisons of differentially expressed transcripts were carried out (ED versus MD, ED versus DB, and MD versus DB) to evaluate the major biological functions, classified into three groups: cellular component (CC), molecular function (MF), and biological process (BP). Blast2GO version 4.0 (Conesa *et al.*, 2005) was used to analyze and to determine significant annotated processes of the main biological functions. A known approach to understanding biological processes of many genes is to identify the metabolic pathways that include the DEGs using KEGG pathway analysis (Kanehisa *et al.*, 2008). For KEGG pathway analysis, all differentially expressed transcripts [between all collection times and in the three comparisons (ED versus MD, ED versus DB, and MD versus DB)] were mapped to terms in KEGG using the DAVID knowledge resource (Huang *et al*., 2008*a*, *b*). In addition, similar transcripts that were differentially expressed in all comparisons in both cultivars were mapped to KEGG pathways to characterize the core gene expression pathways in pear during dormancy. Hence, this group of genes was differentially expressed in ED versus MD, ED versus DB, and MD versus DB in both cultivars. KEGG pathways fulfilling the criterion of a Bonferroni-corrected *P*-value ≤0.05 were defined as significantly enriched.

### Metabolite extraction

Lipid extraction and analysis were performed as previously described in Malitsky *et al.* (2016) and Salem *et al.* (2017) with some modifications: frozen ground bud tissue (100 mg) was mixed with 1 ml of a pre-cooled (–20 °C) homogeneous 1:3 (v/v) methanol:methyl tert-butyl ether (MTBE) mixture containing the following internal standards: 0.1 μg ml^−1^ phosphatidylcholine 34:0 (17:0/17:0) and 0.15 nmol ml^−1^ of LM6002 (Avanti) standard mix. The tubes were vortexed and then sonicated for 30 min in an ice-cold sonication bath (taken for a brief vortex every 10 min). Then, UPLC-grade water:methanol (3:1, v/v) solution (0.5 ml) was added to the tubes followed by centrifugation. The upper organic phase was transferred to a 2 ml Eppendorf tube. The polar phase was re-extracted as described above, with 0.5 ml of MTBE. Both organic phases were combined and dried in a Speedvac and then stored at –80 °C until analysis. For analysis, the dried lipid extracts were resuspended in 250 μl of mobile phase B (see below) and centrifuged again at 13000 rpm and 4 °C for 5 min. The lower polar phase for polar metabolite analysis was stored at –80 °C until use.

### LC-MS for lipidomics analysis

Post-extraction, the supernatant was transferred to an autosampler vial and an aliquot was subjected to UPLC-MS (ultraperformance LC-MS) analysis. Lipid extracts were analyzed using a Waters Acquity UPLC system coupled to a Vion IMS QTOF mass spectrometer (Waters Corp., MA, USA). Chromatographic conditions were as described in Malitsky *et al.* (2016) with slight alterations. Briefly, the chromatographic separation was performed on an Acquity UPLC BEH C8 column (2.1 × 100 mm, i.d., 1.7 μm) (Waters Corp.). Mobile phase A consisted of 45% water (UPLC grade) with 1% 1 M NH_4_Ac, 0.1% acetic acid, and 55% acetonitrile:isopropanol (7:3), with 1% 1 M NH_4_Ac, 0.1% acetic acid (mobile phase B). The column was maintained at 40 °C and the flow rate of the mobile phase was 0.4 ml min^−1^. Mobile phase A was run for 1 min at 100%, then gradually reduced to 25% at 12 min to 0% at 16 min. Then, mobile phase B was run at 100% for 21 min, and mobile phase A was set to 100% at 21.5 min. Finally, the column was equilibrated at 100% A for 25 min. MS parameters were as follows: the source and desolvation temperatures were maintained at 120 °C and 450 °C, respectively. The capillary voltage was set to 3.0 kV and 2 kV for positive and negative ionization mode, respectively; cone voltage was set to 40 V. Nitrogen was used as the desolvation gas and cone gas at a flow rate of 800 l h^−1^ and 30 l h^−1^, respectively. The mass spectrometer was operated in full scan MS^E^ positive resolution mode over a mass range of 50–1800 Da. For the high-energy scan function, a collision energy ramp of 20–60 eV was applied, and for the low-energy scan function, 4 eV was applied. Leu enkephalin was used as a lock-mass reference standard.

### Lipid identification and quantification

LC-MS data were analyzed and processed with UNIFI (Version 1.9.2, Waters Corp.). The putative identification of the different lipid species was performed by comparing accurate mass, fragmentation pattern, ion mobility (CCS) values, and retention time (RT) with an in-house-designed lipid database. Relative levels of lipids were normalized to the internal standards and the amount of tissue used for analysis.

### LC-MS polar metabolite analysis

Metabolic profiling of the polar phase was performed as described in Zheng *et al.* (2015) with minor modifications. Briefly, analysis was performed using the Acquity I class UPLC system combined with a mass spectrometer (Thermo Exactive Plus Orbitrap) which was operated in negative ionization mode. The LC separation was performed using SeQuant Zic-pHilic (150 mm×2.1 mm) with the SeQuant guard column (20 mm×2.1 mm) (Merck). Mobile phase A was acetonitrile and mobile phase B was 20 mM ammonium carbonate plus 0.1% ammonia hydroxide in water. The flow rate was kept at 200 μl min^−1^ and the gradient was: 0–2 min 75% B, 17min 12.5% B, 17.1min 25% B, 19min 25% B, 19.1min 75% B, 19min 75% B.

### Polar metabolite data analysis

Data processing was performed with TraceFinder Thermo Fisher software where detected compounds were identified by retention time and fragments, and verified using the in-house MS library generated by standard injections.

### Metabolite statistical analysis

A total of 174 metabolites were profiled over the five collection times. Metabolite categories were fatty acids (*n*=16), other lipids (*n*=64), and polar metabolites (*n*=94) (Supplementary Table S1). To examine the association between metabolites, for samples of three replicates of five collection times within each cultivar, PCA and a Kendall rank correlation coefficient heat map were plotted using MetaboAnalyst (Xia and Wishart, 2016). The data set was uploaded as a peak intensity table. To evaluate the differential metabolite profiles between collection times and cultivars, ANOVA was carried out with MetaboAnalyst. Significant differences in metabolite profiles were determined according to the FDR (<0.01) value generated with Fisher’s LSD post-hoc analysis. Hierarchical cluster analysis was conducted with JMP to identify similar patterns of metabolite clusters according to dormancy phases.

### Expression of genes underlying QTLs associated with time of vegetative budbreak

Genes were sought within the previously detected VB time QTL regions in LGs 5, 8, 9, 13, and 15 (Gabay *et al.*, 2018). In each QTL peak, two markers that were mapped on the same scaffold were used as flanking markers to identify genes within their interval. *Pyrus* gene data were downloaded from the NCBI database (Wu *et al.*, 2013). Significant transcripts were plotted by MapChart 2.3 with a LOD (logarithm of odds) drop of 1 indicating their genetic mapping and their physical mapping on the pear genome scaffold. The Log2FC graph was plotted with JMP. Pearson correlation was carried out between differentially expressed transcripts, within the previously detected VB time QTLs (Gabay *et al.*, 2018) and the metabolites with significant profile changes. The correlation matrix was performed after filtration using Pearson correlation coefficient >0.8 as a threshold to determine significant correlation (Savoi *et al.*, 2016).

### Integrated biology approach to detect candidate pear bud dormancy regulation genes

The putative candidate genes were selected according their differential expression levels and their correlation to metabolites with significant profile changes during dormancy that were located within QTL intervals. The last stage of sorting was according to KEGG and GO terms. Genes with irrelevant annotation or functions and large SEs were discarded.

## Results

### Determination of dormancy status

The dormancy status was evaluated at six time points of two cultivars that differ greatly in their chilling requirements (Fig. 1). Budbreak percentages at or near 0 were observed on the first two time point dates (1 and 15 December) for both cultivars. At the third collection, ~40% budbreak was observed in both cultivars after being exposed to 816 CUs. Significant differences were observed in the fourth and fifth collections (D) between the cultivars in VB%, where SPD, the low chilling requirement cultivar, showed 50% and 70% budbreak, respectively, and HS, the high chilling requirement cultivar, showed 40% and 50%, respectively (Fig. 1). At the last collection time (E), both cultivars reached 100% VB. However, the first VB date in SPD occurred 3 d earlier than in HS. Three major stages were determined based on budbreak percentage: ED, MD, and DB. Thus, collection times A and B were referred to as ED, C as MD, and D and E as DB.

### RNA-Seq

The biological replicates were highly correlated within each collection time and cultivar (Supplementary Fig. S1). Raw reads (42.7 million) were generated and 96.7% of them (41.2 million reads) were obtained after discarding low-quality reads and adaptor sequences. The clean reads were mapped to the reference *Pyrus* genome (Wu *et al.*, 2013). Successfully mapped reads ranged between 71.6% and 79.3%, and the average was 75.4% (Supplementary Table S2).

### Differential expression analysis

**Fig. 2.**
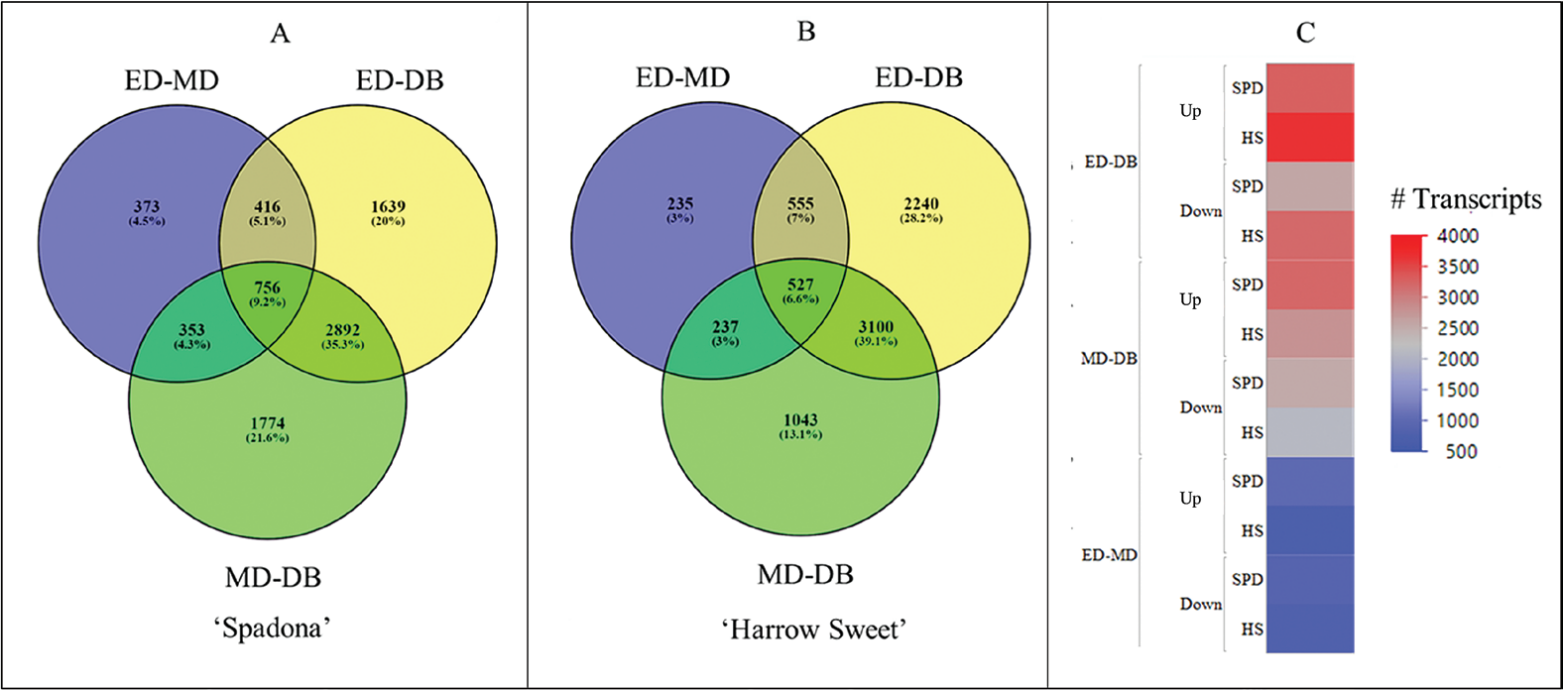
Venn diagrams of significantly differentially expressed transcripts [Log2 fold change values >2 (up-regulated) or < –2 (down-regulated) and *P*_adj_ (FDR) <0.001] at three dormancy phases of pear buds (ED, entry into dormancy; MD, mid-dormancy; DB, dormancy break). (A) Low chilling requirement cv. Spadona (SPD). (B) High chilling requirement cv. Harrow Sweet (HS). (C) The number of transcripts that were up- and down-regulated in each of the comparisons for both SPD and HS.

According to DESeq2 (Love *et al.*, 2014) analysis [Log2FC >2 (up-regulated) or < –2 down-regulated and *P*_adj_ <0.001], a total of 8771 transcripts were differentially expressed in HS and 8785 transcripts in SPD in the collection time comparisons (i.e. times A–E). According to the comparison of the three major stages ED–MD, ED–DB, and MD–DB (i.e. excluding comparisons A–B and D–E), we observed the highest number of differentially expressed transcripts in HS (*n*=6442) between ED and DB, and in SPD between MD and ED (*n*=5775). However, in SPD, we observed almost the same number of differentially expressed transcripts between ED and DB (*n*=5703). In both cultivars, the lowest number of differentially expressed transcripts was observed between ED and MD, 1898 in SPD and 1554 in HS (Fig. 2A, B). There was a higher number of up-regulated versus down-regulated transcripts in all dormancy stage comparisons in both cultivars, except for ED versus MD in HS, where more down-regulated (*n*=798) than up-regulated (*n*=756) transcripts were observed (Fig. 2C).

### GO annotations and KEGG enrichment analysis of differentially expressed transcripts

Transcripts belonging to the BP category constituted most of the GO annotations in SPD in MD versus DB (23940 GO annotations) and in ED versus MD (*n*=6017) comparisons, and in HS, most BP transcripts were observed in ED versus DB (23006 GO annotations). Minor functional activity was observed for all of the biological functions in ED versus MD in HS, indicating little difference in gene expression between the beginning (ED) and middle of dormancy (MD) in this high chilling requirement cultivar. ‘Metabolic process’ was the most notable subcategory of BP in all comparisons for both cultivars (Supplementary Fig. S2).

**Fig. 3.**
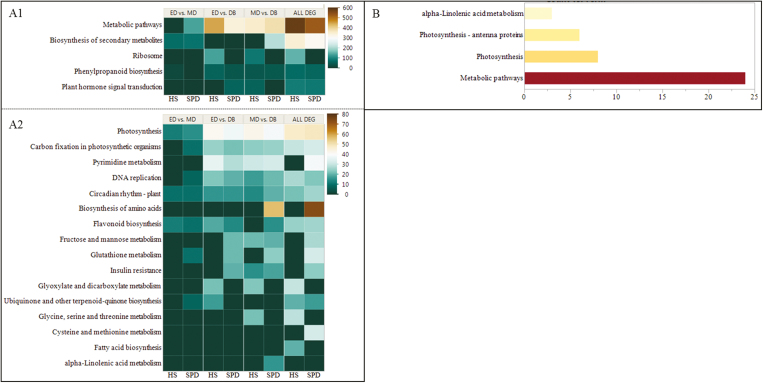
Differentially expressed genes (DEGs) in enriched KEGG pathways in three main dormancy phases (ED, entry into dormancy; MD, mid-dormancy; DB, dormancy break) of two European pear cultivars differing in their chilling requirements (HS, high chilling requirement cv. Harrow Sweet; SPD, low chilling requirement cv. Spadona). (A) The number of significantly DEGs in the most enriched KEGG pathways between three stages of dormancy. Significance level was determined according to the modified Fisher exact *P*-value at <0.05, for which gene enrichment analysis was considered strongly enriched in the annotation categories. (A1) Number of transcripts per KEGG pathway=0–600. (A2) Number of transcripts per KEGG pathway=0–80. Color indicates the number of transcripts related to enriched pathways. (B) Number of significant common DEGs in all three comparisons and two cultivars in the significantly enriched KEGG pathways.

The most enriched KEGG pathway in all comparisons (*n* >100), as well as for all differentially expressed transcripts (*n* ~500), was ‘metabolic pathways’ in SPD, and in MD versus DB (*n* ~350) and for all differentially expressed transcripts (*n* >500) in HS (Fig. 3A). In both cultivars, MD versus DB comparison contained the highest total number of enriched pathways, indicating that most processes occur between mid-dormancy and dormancy break. However, in SPD, more enriched pathways were observed in ED versus DB and ED versus MD, indicating that in this low chilling requirement cultivar, processes start at the beginning of dormancy and earlier than in the high chilling requirement cultivar HS (Fig. 3A1, A2). The KEGG pathways analysis of the common DEGs between the two cultivars among all three comparisons revealed three general categories, including ‘metabolic pathway’ and ‘photosynthesis’, and only one specific pathway, ‘alpha-linolenic acid’ (Fig. 3B).

### Metabolic analysis

**Fig. 4.**
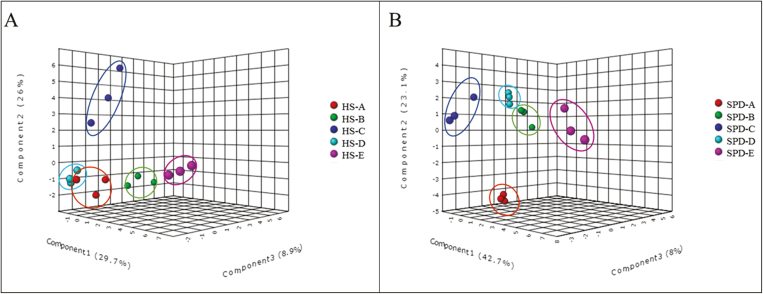
PCA of three biological replicates from five collection time points (A–E) was conducted using relative values of 174 metabolites during the dormancy phases of (A) high chilling requirement cv. Harrow Sweet (HS) and (B) low chilling requirement cv. Spadona. The three-dimensional plot (components 1–3) shows that replicates are assigned to five main groups according to their collection time (indicated by circles).

**Fig. 5.**
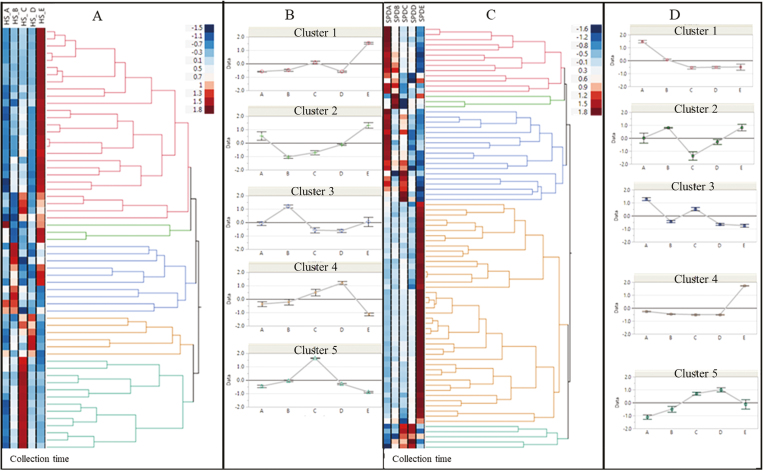
Hierarchal clustering of significant (FDR <0.01 by Fisher’s LSD post-hoc analysis) changes in metabolite content between collection times. (A) Metabolite clusters of high chilling requirement cv. Harrow Sweet (HS). (B) Means of metabolite cluster normalized content profiles in HS. (C) Metabolite clusters of low chilling requirement cv. Spadona (SPD). (D) Means of metabolite cluster normalized content profiles in SPD. Colors indicate the normalized value of metabolite contents at each collection time: dark red, high content; dark blue, low content. The full list of the metabolites which are divided into clusters and their relative accumulation levels is presented in Supplementary Table S3.

According to Kendall rank correlation (Supplementary Fig. S3) coefficient analysis, there was a clear division between collection times in the metabolic profiles in both cultivars. The PCA supported these results, except for HS, sample A3 (collection time A), whose correlation to the other replicates was lower (Fig. 4). We putatively identified 59 metabolites in HS and 82 in SPD whose profiles were significantly different (FDR <0.01) between at least two collection times (Supplementary Table S3). Forty-four metabolites displayed differential levels in both cultivars, although the patterns of the changes differed between the cultivars; that is, the same metabolites displayed different patterns of significant changes in content during dormancy (Fig. 5). Fifteen metabolites were observed only in HS. Most of the metabolites that were observed only in HS were phospholipids (*n*=6) and organic acids (*n*=4); 38 metabolites were observed only in SPD, among which six were fatty acids and five were sugars (Supplementary Table S3).

### Hierarchical cluster analysis of metabolic profiles

Five metabolite clusters were identified in each cultivar, when at least one metabolite had a significantly differential (FDR <0.01) profile between at least two collection time points (Fig. 5). Cluster #4 in HS included six metabolites, four of them sugars (Supplementary Table S3). The metabolite accumulation pattern of this cluster was similar to that of cluster #5 in SPD, which contained only sugars (Supplementary Table S3). However, in this cluster, the decrease in metabolite levels between collections D and E was sharper than in cluster 5 of SPD. Cluster #5 in HS (*n*=13) showed an increase toward MD and then a decrease toward DB; nine phospholipids were included in this cluster, which was the biggest subcategory group (Fig. 5A; Supplementary Table S3). In cluster #4 in SPD, the metabolite levels were low during all dormancy stages and increased sharply toward the last collection time when higher percentages of budbreak were observed; five of seven fatty acids displaying significant differences in their levels were in this cluster (Fig. 5C, D). Cluster #5 in SPD included only sugars, with low levels that increased toward MD and collection time D, then decreased toward the last collection time (Supplementary Table S3).

### Expression of genes underlying QTLs related to timing of vegetative budbreak

**Fig. 6.**
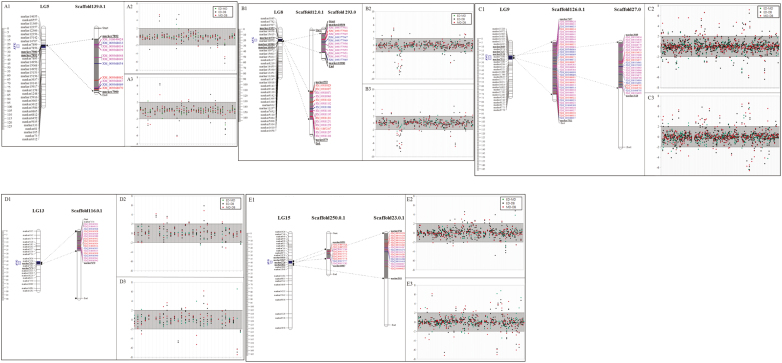
Expression of genes underlying the main genotype QTL detected in linkage group (LG) 5 (A), LG8 (B), LG9 (C), LG13 (D), and LG15 (E) in Gabay *et al.*, 2018. (1) QTL genetic position (LGs) and physical position on the pear scaffold. Flanking markers on both physical and genetic maps are in bold. Differentially expressed genes are marked in blue, red, and pink. (2) Expression distribution of the genes underlying the QTL in HS. (3) Expression distribution of the genes underlying the QTL in SPD. Genes with Log2FC (*y*-axis in 2 and 3) lower than –2 or higher than 2 were considered differentially expressed. The type of comparison is indicated in the key: ED versus MD in green, ED versus DB in black, and MD versus DB in red. Gene data are available in Supplementary Table S4.

Candidate genes were sought within the overall mean QTL region in LGs 5 (Fig. 6A), 8 (Fig. 6B), 9 (Fig. 6C), 13 (Fig. 6D), and 15 (Fig. 6E) which were detected in Gabay *et al.* (2018). Within the major QTL interval (genetic position: 18.4–22.4 cM) in LG8, explaining 28% of the phenotypic variance, we identified a total of 92 transcripts on scaffold 293.0 between the flanking markers 10954 and 10980, and on scaffold 12.0.1 between the flanking markers 1552 and 1579 (Fig. 6B). We were able to generate expression data on 84 of the 90 transcripts located within this QTL interval; 23 transcripts of 19 genes were differentially expressed (Log2FC >2). *PcDAM1* and *PcDAM2*, putative orthologs of *PpDAM1* and *PpDAM2*, transcript variants were found with the highest Log2FC in both cultivars. XM_009377952 in HS (largest Log2FC= –6.25, in collection time B versus E, i.e. ED versus DB) and XM_009377951 in both cultivars (HS, largest Log2FC= –7, ED versus MD; SPD, largest Log2FC= –6.85, ED versus DB) were sharply down-regulated between the dormancy phases. Both transcripts encoded the MADS-box protein-encoding *AGL24-like* gene (LOC103964948), *PcDAM1*. The XM_009377955 transcript variant of the MADS-box protein-encoding *AGL24-like* gene (gene name: *PcDAM2*) was significantly down-regulated between collection times B and E (i.e. ED versus DB (Log2FC for HS= –8.8, for SPD= –7.48)]. Two transcripts encoding the MADS-box gene *AGL24-like* (LOC103964952) had major differences in their expression levels in the comparison of ED versus DB (XM_009377956, Log2FC, HS= –7.9, SPD= –8.5, XM_009377958, Log2FC HS= –6.1, SPD= –6.48) (Supplementary Table S4). Transcript variants of *FT-interacting protein 1-like* had large significant differences in the comparison between ED and DB (Log2FC, HS= –5.26, SPD= –4.01) and MD versus DB (Log2FC, HS= –5.15, SPD= –3.94). The QTL interval (*R*^2^=9.8%) in LG9 contained 194 transcripts on two scaffolds—126.0.1 and 27.0 (Fig. 6C); 62 transcript variants of 54 genes were differentially expressed (Log2FC >2). The most extreme difference in expression in SPD was observed with XM_009366925 (Log2FC=6.62) and XM_009366926 (Log2FC= –6.61) in MD versus DB, both variants of histidine kinase 2 (LOC103955055) associated with the plant hormone signaling pathway. Those variants showed major differential expression in HS as well (XM_009366925 Log2FC= –6.1, ED versus DB and XM_009366926 Log2FC=7.17, MD versus DB). Specifically in HS, a great difference in expression level was detected for the XM_009366841 (Log2FC=6.62, ED versus DB) transcript encoded by the gene for palmitoyl-monogalactosyldiacylglycerol delta-7 desaturase, chloroplastic-like (LOC103954983), which is associated with biosynthesis of unsaturated fatty acids and fatty acid metabolism pathways. Within the additional QTL intervals in LG5 (*R*^2^=5.2%), LG13 (*R*^2^=4.2%), and LG15 (*R*^2^=6), we detected 8, 12, and 24 DEGs, respectively. Expression of transcripts underlying the five QTLs is presented in Supplementary Table S4.

**Fig. 7.**
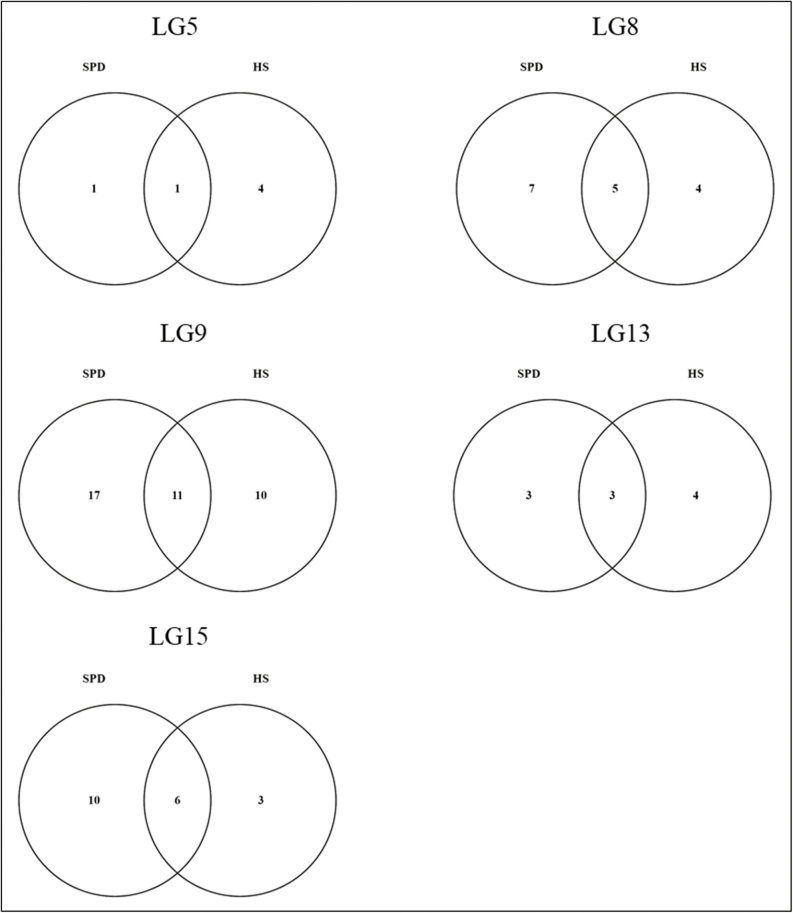
Venn diagrams showing the numbers of differentially expressed transcripts underlying the detected QTLs in Gabay *et al.* (2018) correlated (>0.8) to significant changes in metabolites during dormancy in vegetative buds of European pear. LG=linkage group of the detected QTLs in Gabay *et al.* (2018). Numbers indicate the specific and common transcripts differentially expressed in two cultivars. HS=Harrow Sweet, high chilling requirement cultivar. SPD=Spadona, low chilling requirement cultivar.

Transcript and gene data within the QTL intervals and their correlation to the significant changes in metabolite content are presented in Supplementary Table S5; 37 transcripts were correlated to SPD metabolites, 26 to HS metabolites, and 25 to both. The number of differentially expressed transcripts per LG ranged between 6 and 38 (Fig. 7).

### Characterization of candidate genes and identification of new putative candidate genes

**Fig. 8.**
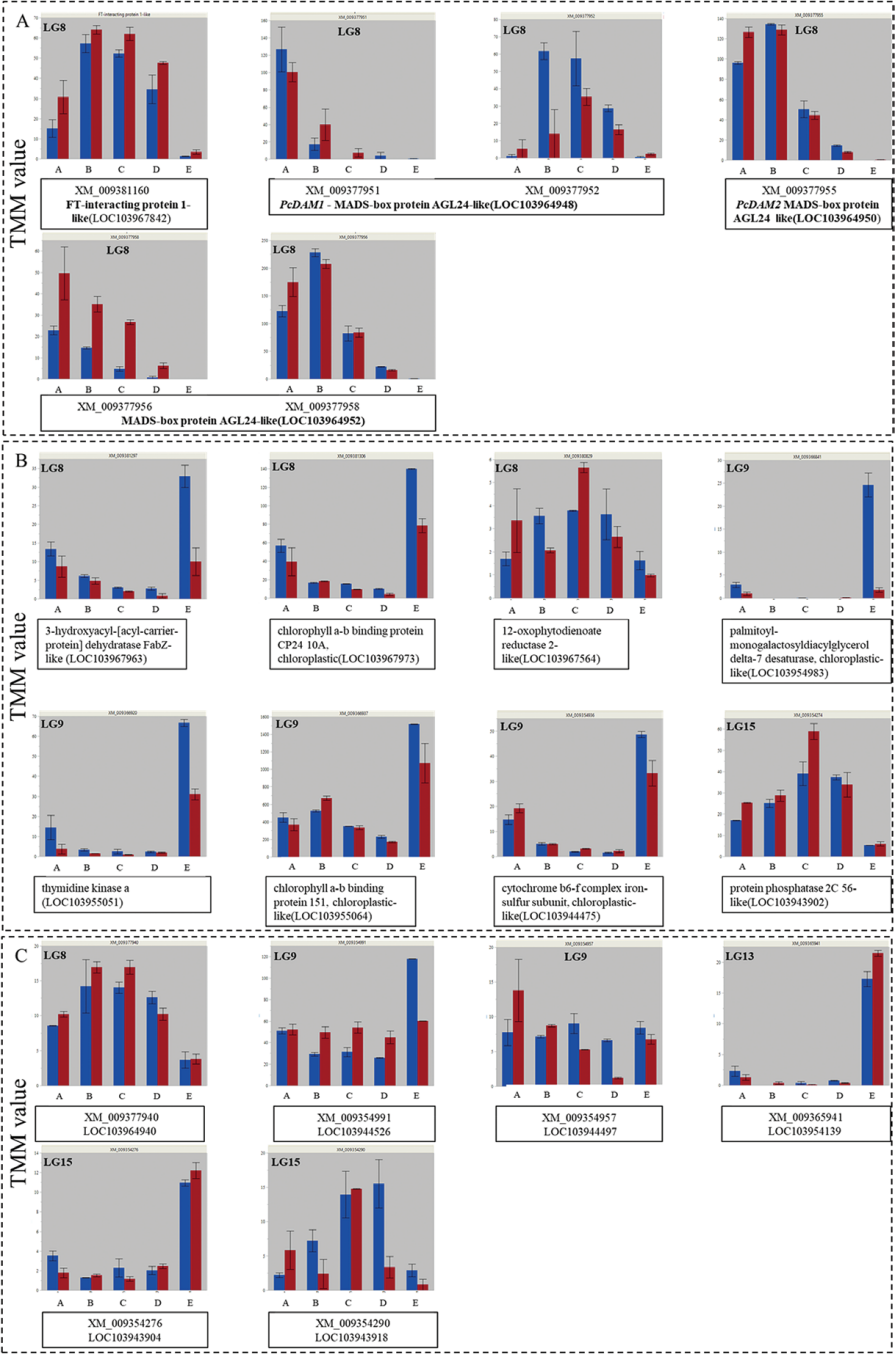
Expression of selected putative candidate genes. (A) Transcription factor genes. (B) Genes associated with metabolic pathways and plant hormone signal transduction. (C) Genes with unknown function. The *y*-axis represents expression values, trimmed mean of M values (TMM)-normalized counts generated by the RNA-Seq. The *x*-axis represents the collection time. Gene name, transcript index, and gene ID are indicated in-frame below the gene expression graphs. Gene linkage group (LG) is indicated in the upper left corner of the frames, except for (A), which shows genes in LG8. Red bars, SPD gene expression TMM values; blue bars, HS gene expression TMM values.

In total, we obtained 106 DEGs (144 transcripts) underlying the QTLs between dormancy phases: 89 transcripts (encoded by 72 genes) were found correlated (*R*^2^ >0.8) to one or more metabolites with significant changes during dormancy. Out of 106 genes, eight genes were related to metabolic pathways and plant hormone signal transduction, including the α-linolenic acid-related gene, *12-oxophytodienoate reductase 2-like* (LOC103967564), and four transcription factor genes, such as MADs-box, *PcDAM1* and *PcDAM2*. In addition, using the same integrated approach, we were able to identify six new putative candidate genes that, to date, have a non-characterized function. Expression of the putative candidate genes during dormancy is presented in Fig. 8.

## Discussion

### Dormancy phase transitions and differences between high and low chilling requirement cultivars

Although dormancy has been characterized by extreme changes in metabolic profile in fruit trees (Wang and Faust, 1990; Izadyar and Wang, 1999; Del Cueto *et al.*, 2017; Ionescu *et al.*, 2017), very little is known about the metabolic profile in pear, and specifically European pear dormancy. In this study, we focused on the major metabolite groups as described in other fruit species to create a correlation with chilling accumulation and dormancy break; among these were unsaturated fatty acids (Erez *et al.*, 1997), sugars (Falavigna *et al.*, 2018), and phospholipids (Izadyar and Wang, 1999).

The comparative transcriptome analysis aimed to reveal differential gene expression between the three major stages of dormancy: the first stage of dormancy (ED) with exposure to CUs (i.e. collection times A and B), the second stage of dormancy establishment (MD) (i.e. collection time C), and dormancy break (DB) (i.e. collection times D and E). In addition, the differences in gene expression, GO terms, and KEGG pathways cultivars were analyzed. When we compared all differentially expressed transcripts between SPD and HS, we found similarities in the KEGG pathways, with ‘metabolic pathways’ being the most enriched (Fig. 3). Hence, most of the differentially expressed transcripts between the different dormancy phases were associated with metabolic processes. This indicates the importance of gene regulation of metabolic processes during the transitions between dormancy phases, and supports earlier results in Asian pear (Liu *et al.*, 2012; Bai *et al.*, 2013). The comparison between the cultivars revealed that many of the biological processes in the GO analysis occur later in HS than in SPD. Hence, SPD reacted faster to CU accumulation and many of the biological processes, such as ‘metabolic process’ and ‘biosynthetic process’, were observed in SPD in ED versus MD, whereas in HS they occurred in ED versus DB or in MD versus DB (Supplementary Fig. S2).

### α-Linolenic acid and other fatty acid profile changes in European pear buds during dormancy

We detected a total of 22 DEGs (7 specific to SPD, 4 specific to HS, and 11 common) related to the α-linolenic acid pathway according to KEGG in our transcriptome analysis of two cultivars that differ greatly in their chilling requirements. Several DEGs related to JA pathways were also observed (Supplementary Table S6). JA is synthesized by α-linolenic acid (Hu *et al.*, 2013) and suggested to have a key role in dormancy break regulation (Taniguchi *et al.*, 2003). Moreover, KEGG analysis of the common DEGs between all three time comparisons and two cultivars showed that the α-linolenic acid pathway was the only specific significantly enriched KEGG pathway (Fig. 3B). In addition, a gene-related to α-linolenic acid, 12-oxophytodienoate reductase 2-like (LOC103967564), is located within the major QTL interval in LG8 that was identified in Gabay *et al.* (2018). Indeed, we observed significantly sharp increase in α-linolenic acid content toward the end of dormancy in both cultivars (Fig. 5A, C; Supplementary Table S3). However, in SPD, we observed six other unsaturated fatty acids, including linoleic acid, with significant changes during dormancy. In addition, our differentially expressed transcript analysis reveals several genes that are related to the metabolic pathway of linolenic acid (Supplementary Table S6). Four fatty acids were in the same cluster as α-linolenic acid (cluster 4; Supplementary Table S3) which was characterized by low content during all dormancy stages and a sharp increase toward dormancy break (Fig. 5C, D). The level of fatty acids is directly correlated with chilling accumulation (Erez *et al.*, 1997). Although both cultivars were exposed to the same number of CUs, we observed different fatty acid profiles during dormancy. Therefore, we suggest a potential role for α-linolenic acid that was detected by all the methods that were used in this study, and for the six other fatty acids—lauric acid, linoleic acid, margaric acid, nonadecylic acid, palmitic acid, and stearic acid—in the changes in membrane metabolite composition that allow budbreak to occur, and in representing differences between low and high chilling requirements. Although, we did not observe a large set of differentially expressed transcripts that are associated with lauric acid, margaric acid, nonadecylic acid, palmitic acid, and stearic acid biosynthesis or metabolism, the changes in their content were dramatically changed between the dormancy phases in the low chill requirement cultivar, ‘Spadona’, and their role should be further examined.

### Sugar and phospholipid profiles during bud dormancy

We found significant content changes for 11 sugars in SPD and 8 in HS. In both cultivars, the changes in raffinose content followed a similar pattern (cluster 4 in HS and cluster 5 in SPD; Fig. 5). Raffinose accumulation has been observed in apple during dormancy, and it is suggested to play a role in protecting buds against drought (Falavigna *et al.*, 2018); this may also be valid here, due to the patterns of accumulation toward budbreak, after the trees were transferred from the controlled room and regular irrigation. However, we obtained other sugars, such as sucrose, within the same clusters (Fig. 5). Sugars are essential for bud growth regulation (Roitsch and González, 2004), and spring budbreak is greatly affected by sugar availability (Tixier *et al.*, 2017). Therefore, we assume that sugar accumulation signals sufficient chilling accumulation, allowing budbreak as soon as favorable climate conditions arise, as suggested recently in grape (Khalil-Ur-Rehman *et al.*, 2017*b*). The content of most phospholipids increased toward dormancy establishment (collection time C) in HS, cluster 5 (Fig. 5A, B; Supplementary Table S3), coinciding with peach bud dormancy, where a significant increase in phospholipids was observed with chilling accumulation (Erez *et al.*, 1997). However, we did not observe the same patterns for SPD phospholipids (clusters 1 and 3; Fig. 5C, D; Supplementary Table S3). The majority of phospholipids in SPD with significant changes in content were observed in cluster 3, where an elevated content was observed at dormancy establishment as in HS, cluster 5. However, the content of most phospholipids was higher at the beginning of dormancy compared with HS. Hence, the phospholipids accumulated earlier in SPD with a low chilling requirement. Therefore, we suggest that phospholipids are being accumulated in response to chilling exposure, which occurs earlier in low chilling requirement cultivars.

### Differentially expressed putative candidate genes and co-localization with QTLs

DEGs were co-localized with the previously identified QTLs that are associated with VB time (Gabay *et al.*, 2018). Some of the genes that are associated with dormancy and VB are likely to be positioned outside the QTL interval. For instance, if SPD and HS carry the same alleles for a gene that regulates dormancy, our QTL analysis might not detect this particular gene since no segregation could be observed. However, QTL detection can lead to gene detection in plants such as tomato (Frary *et al.*, 2000) and rice (Sallaud *et al.*, 2003). The genes that underlie the QTLs and were differentially expressed included *PcDAM1* and *PcDAM2*, whose expression differs between dormancy stages in Japanese pear (Saito *et al.*, 2013, 2015) and apple (Mimida *et al.*, 2015). To the best of our knowledge, the differential expression levels of *DAM* genes during dormancy are reported here for the first time in European pear, which supports our approach since their association with dormancy was previously detected in other fruit trees. Using the same approach of candidate gene detection, we found *FT-interacting protein 1-like* (LOC103967842) which supports the major role of *FT* genes as dormancy regulators (Niu *et al.*, 2016). *FT-interacting protein 1* is an important factor for transportation of FT protein in Arabidopsis. Late flowering was observed when *FT-interacting protein 1* was non-functional in Arabidopsis (Liu *et al.*, 2012). Transcription factor genes were differentially expressed within cultivars between different collection times and between cultivars at the same time. The differential expression level between cultivars was observed in *FT-interacting protein 1-like* at collection times A, C, and D and in *PcDAM2* at collection times A and D. In *PcDAM1* and *MADS-box protein AGL24-like* (LOC103964952), we observed differences between the cultivars in the variant expression patterns of the transcripts and in the expression of the same transcript variant (Fig. 8A). Therefore, we suggest that those genes may play a role in the determination of chilling requirements and the regulation of the content of metabolites at different dormancy phases. The interaction between *DAM* and *FT* genes was suggested to be due to the correlation of the expression levels of these genes (Horvath *et al*., 2010; Ubi *et al.* 2010; Yamane *et al*., 2011). However, in Japanese pear, no correlation between *PpDAM* genes and *PpFT1a/PpFT2a* was observed (Bai *et al*, 2013). The expression levels, in this study, of *FT* related-genes and the *DAM* genes show that the highest expression levels of *FT-interacting protein 1* were observed at the same time or later than those of *PcDAM1* and *PcDAM2*. However, these results alone cannot imply a regulatory role for *DAM* genes for the activation of the *FT* genes as indicated in leafy spurge (Horvath *et al*., 2008) and therefore a functional study is needed. In addition, we identified important genes related to metabolic pathways according to the KEGG pathway (Fig. 8). The 12-oxophytodienoate reductase 2-like (LOC103967564) gene, which is directly related to both α-linolenic acid and linoleic acid pathways, is located in the major QTL in LG8. An Arabidopsis homolog gene, 12-oxophydoienoic acid reductase 3 (*opr3*), mutant reduced JA synthesis in Arabidopsis (Smith and Zhao, 2016).

Interestingly, six uncharacterized DEGs with unknown function were found within the QTL interval and are suggested here for the first time as candidate genes associated with dormancy regulation (Fig. 8). In addition, due to the known role of the plant hormones, ABA and JA (Taniguchi *et al.*, 2003; Ophir *et al.*, 2009; [Bibr CIT0003][Bibr CIT0043]), and to the role of linoleic acid and α-linolenic acid in dormancy regulation, the full list of the differentially expressed transcripts that are related to those factors is provided in Supplementary Table S6. We observed 27 differentially expressed transcripts that are associated with the ABA pathway, 22 with α-linolenic acid, 21 with linoleic acid, and 8 with JA according to KEGG. DEGs that are related to other plant hormones, such as gibberellin and ethylene that were correlated with the dormancy status in Bai *et al.* (2013), were not detected on a large scale in this study.

### Conclusion

**Fig. 9.**
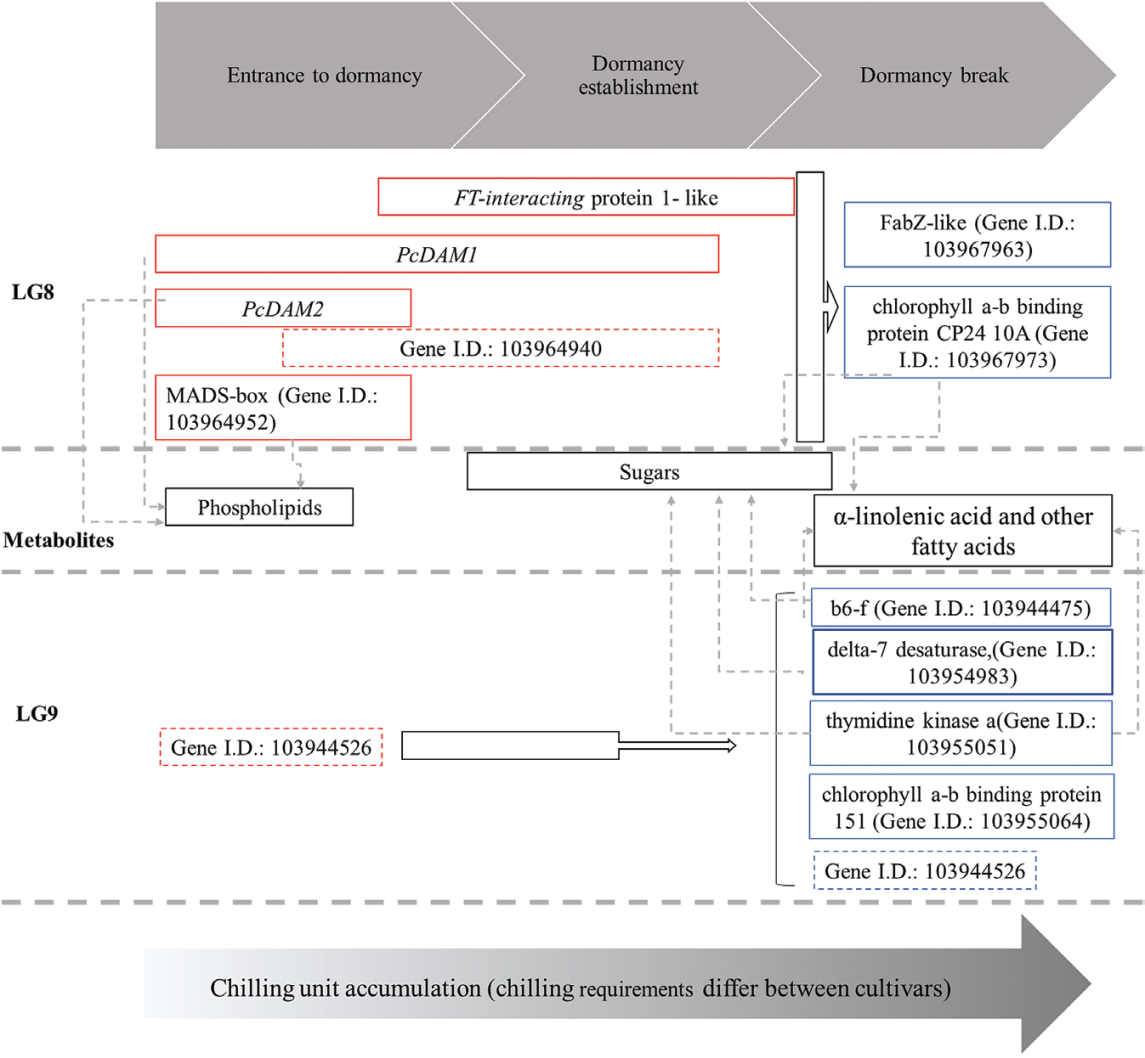
Suggested model of dormancy regulation by genes underlying the two most significant QTLs in LG8 and LG9 considering the metabolite profiles of the buds during dormancy. White block arrows indicate possible gene by gene regulation. Dotted arrows indicate strong correlation (*R*^2^>0.95) between gene expression and metabolite content. The sizes of the frames of both metabolites and genes represent the time, according to the time frame across the top of the figure, of the highest expression level (gene) or relative accumulation level (metabolite). Red frames indicate transcription factor genes. Blue frames indicate genes related to metabolic pathways based on KEGG enrichment. Dotted frames indicate uncharacterized genes and their suggested function according to color.

The suggested candidate genes of European pear bud dormancy regulation in this study support earlier studies on *DAM* and *FT* genes (Bai *et al.*, 2013; Mimida *et al.*, 2015; Niu *et al.*, 2016). In addition, specific candidate genes that are associated with metabolic pathways are suggested here for the first time, along with uncharacterized candidate genes with unknown function (Fig. 8). Our suggested model of dormancy regulation (Fig. 9) considers the changes in metabolic profile. These results highlight the effect of α-linolenic acid and *DAM* genes in dormancy regulation in European pear. We assume that at the beginning of dormancy, phospholipids are accumulated along with CUs and may be needed for sugar biosynthesis. Then sugars are accumulated and may signal sufficient chilling accumulation, opening the way for budbreak, as reported for grapes (Khalil-Ur-Rehman *et al.*, 2017*b*). The last phase of dormancy is characterized by an increase in fatty acids which causes membrane changes represented by a different metabolite composition, allowing budbreak to occur. In this study, we did not examine the level of hormones regarding the dormancy status; however, since the role of plant hormones in pear dormancy is known (Taniguchi *et al.*, 2003; Ophir *et al.*, 2009; Bai *et al.*, 2013; Li *et al.*, 2018) and since α-linoleic acid is a JA precursor (Eremina *et al.*, 2016), we suggest examining those levels, and especially JA levels, in a future study. We focused on the genes underlying the most significant QTLs (LG8 and LG9) in Gabay *et al.* (2018) in our suggested model. Transcription factors such as DAM are mostly expressed at the beginning of and in mid-dormancy. Therefore, we hypothesize that these genes signal to the tree buds the entry into dormancy when the temperature is decreasing, by activating genes that are related to metabolic pathways and regulate the synthesis of metabolites that are essential for bud survival during dormancy and budbreak in the spring. To examine whether the transcription factors activate the metabolic pathway genes and the possible effect on metabolite content level, further research is needed.

## Supplementary data

Supplementary data are available at *JXB* online.

Table S1. Data on metabolites profiled during bud dormancy.

Table S2. Number of reads based on RNA-Seq data per sample.

Table S3. Significant metabolite content changes between collection times.

Table S4. Transcript expression data of genes within the QTLs.

Table S5. R Pearson correlation between DE transcripts within the identified QTLs and metabolites with significant changes.

Table S6. Differentially expressed transcripts that are associated with key fatty acids and plant hormone pathways.

Fig. S1. PCA plot of RNA-Seq samples.

Fig. S2. GO annotation counts of differentially expressed transcripts between dormancy phases.

Fig. S3. Heat map based on Kendall rank correlation coefficient between the metabolite replicates on different collection dates.

Supplementary Tables S1-S6

Supplementary Figures-S1-S3
