# Supplementary material for: Transcriptome analysis and metabolic profiling reveal the key role of α-linolenic acid in dormancy regulation of European pear
Source: J Exp Bot. 2018 Dec 26;70(3):1017–31. doi: 10.1093/jxb/ery405 (PMC6363095; doi:10.1093/jxb/ery405)
Supplement: Supplementary Figures-S1-S3 [file ery405_suppl_supplementary-figures-s1-s3.pdf]

Supplementary Figures

Figures Legends

Fig. S1. Principle Component Analysis (PCA) of the RNA-Seq samples. Legend indicates collection times (i.e., A–E) and cultivar (HS = high-chilling-requirement cv. Harrow Sweet, SPD = low-chilling-requirement cv. Spadona).

Fig. S2. GO annotation counts of transcripts that were differentially expressed between three dormancy phases: entrance to dormancy (ED), middle of dormancy (MD), and dormancy break (DB). GO categories that were significantly enriched (FDR < 0.05) were analyzed with level of significance in pairwise comparisons (ED vs. MD, ED vs. DB, MD vs. DB). Transcripts were annotated into three main categories: (A) biological process, (B) cellular component, and (C) molecular function. The number of transcripts annotated with each GO term is indicated in the color key.

Fig. S3. Heat map based on Kendall rank correlation coefficient between replicates of collection time (A–E) in two cultivars (HS = high-chilling-requirement cv. Harrow Sweet, SPD = low-chilling-requirement cv. Spadona). Dark red indicates a high correlation and dark blue a low correlation between samples.

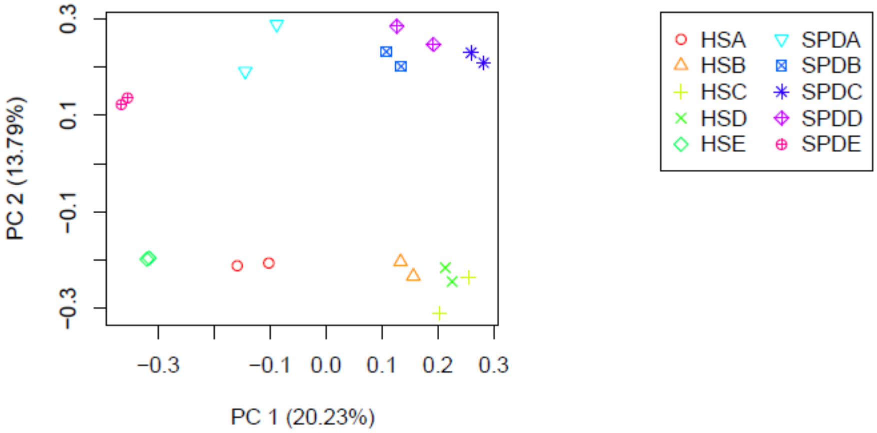

PC 2 (13.79%)
0.3
0.1
−0.1
−0.3
−0.3
−0.1
0.0
0.1
0.2
0.3
PC 1 (20.23%)
HSA
HSB
HSC
HSD
HSE
SPDA
SPDB
SPDC
SPDD
SPDE

Fig. S1.

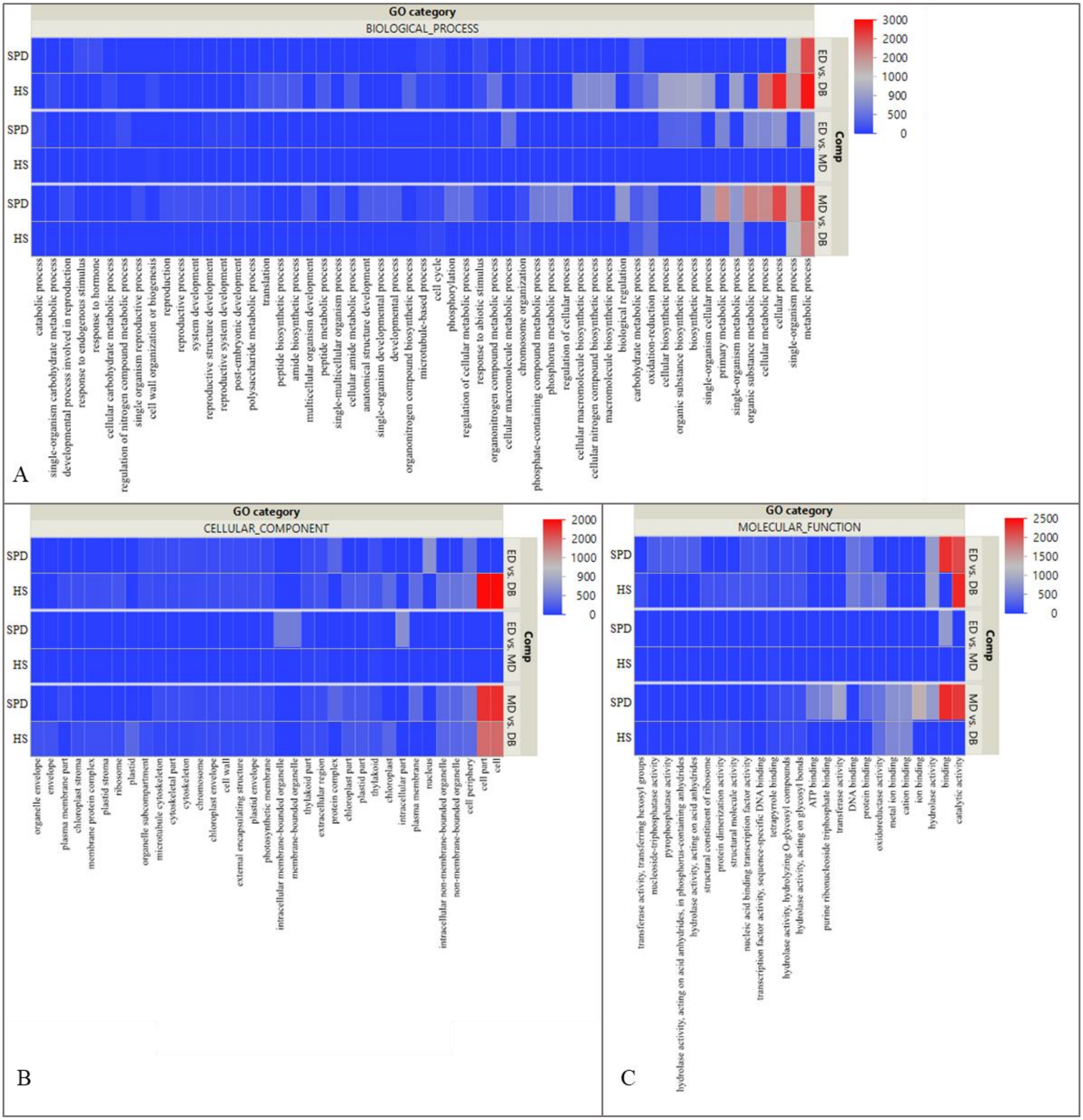
A
GO category
BIOLOGICAL_PROCESS
SPD
HS
SPD
HS
SPD
HS
ED vs. DB
ED vs. MD
MD vs. DB
Comp
3000
2000
2000
1000
900
500
0
catabolic process
single-organism carbohydrate metabolic process
developmental process involved in reproduction
response to endogenous stimulus
response to hormone
cellular carbohydrate metabolic process
regulation of nitrogen compound metabolic process
single organism reproductive process
cell wall organization or biogenesis
reproduction
reproductive process
system development
reproductive structure development
reproductive system development
post-embryonic development
polysaccharide metabolic process
translation
peptide biosynthetic process
amide biosynthetic process
multicellular organism development
peptide metabolic process
single-multicellular organism process
cellular amide metabolic process
anatomical structure development
single-organism developmental process
developmental process
organonitrogen compound biosynthetic process
microtubule-based process
cell cycle
phosphorylation
regulation of cellular metabolic process
response to abiotic stimulus
organonitrogen compound metabolic process
cellular macromolecule metabolic process
chromosome organization
phosphate-containing compound metabolic process
phosphorus metabolic process
regulation of cellular process
cellular macromolecule biosynthetic process
cellular nitrogen compound biosynthetic process
macromolecule biosynthetic process
biological regulation
carbohydrate metabolic process
oxidation-reduction process
cellular biosynthetic process
organic substance biosynthetic process
biosynthetic process
single-organism cellular process
primary metabolic process
single-organism metabolic process
organic substance metabolic process
cellular metabolic process
cellular process
single-organism process
metabolic process
B
GO category
CELLULAR_COMPONENT
SPD
HS
SPD
HS
SPD
HS
ED vs. DB
ED vs. MD
MD vs. DB
Comp
2000
2000
1000
900
500
0
organelle envelope
envelope
plasma membrane part
chloroplast stroma
membrane protein complex
plastid stroma
ribosome
plastid
organelle subcompartment
microtubule cytoskeleton
cytoskeletal part
cytoskeleton
chromosome
chloroplast envelope
cell wall
external encapsulating structure
plastid envelope
photosynthetic membrane
intracellular membrane-bounded organelle
membrane-bounded organelle
thylakoid part
extracellular region
protein complex
chloroplast part
plastid part
thylakoid
chloroplast
intracellular part
plasma membrane
nucleus
intracellular non-membrane-bounded organelle
non-membrane-bounded organelle
cell periphery
cell part
cell
C
GO category
MOLECULAR_FUNCTION
SPD
HS
SPD
HS
SPD
HS
ED vs. DB
ED vs. MD
MD vs. DB
Comp
2500
2000
1500
1000
500
0
transferase activity, transferring hexosyl groups
nucleoside-triphosphatase activity
pyrophosphatase activity
hydrolase activity, acting on acid anhydrides, in phosphorus-containing anhydrides
hydrolase activity, acting on acid anhydrides
structural constituent of ribosome
protein dimerization activity
structural molecule activity
nucleic acid binding transcription factor activity
transcription factor activity, sequence-specific DNA binding
tetrapyrrole binding
hydrolase activity, hydrolyzing O-glycosyl compounds
hydrolase activity, acting on glycosyl bonds
ATP binding
purine ribonucleoside triphosphate binding
transferase activity
DNA binding
protein binding
oxidoreductase activity
metal ion binding
cation binding
ion binding
hydrolase activity
binding
catalytic activity

Fig. S2.

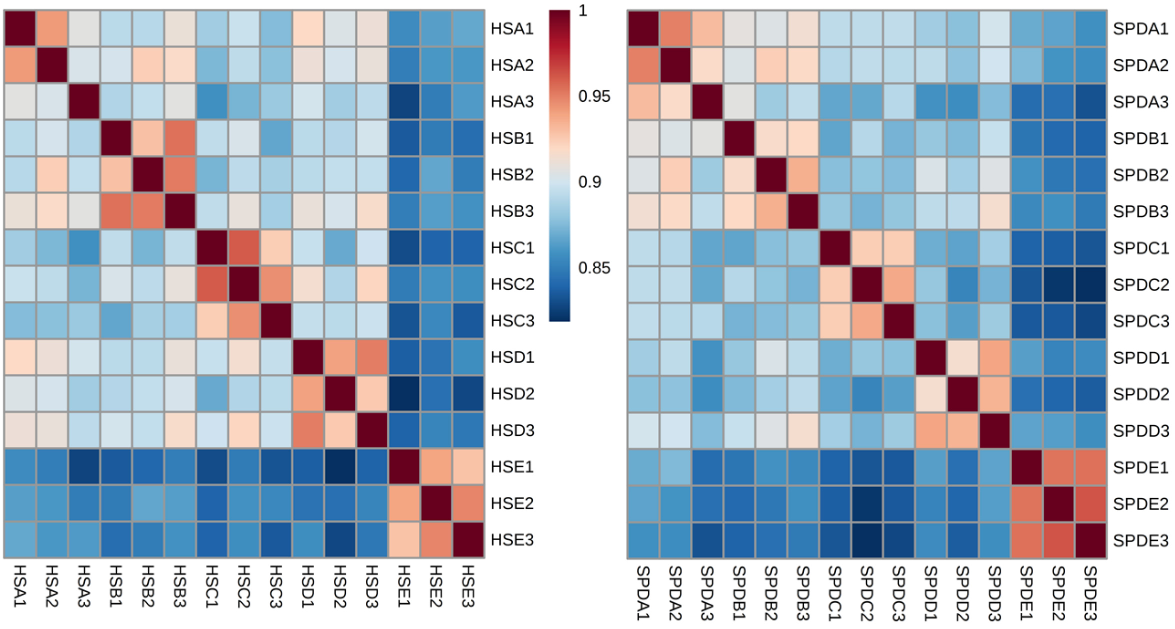

HSA1
HSA2
HSA3
HSB1
HSB2
HSB3
HSC1
HSC2
HSC3
HSD1
HSD2
HSD3
HSE1
HSE2
HSE3
1
0.95
0.9
0.85
SPDA1
SPDA2
SPDA3
SPDB1
SPDB2
SPDB3
SPDC1
SPDC2
SPDC3
SPDD1
SPDD2
SPDD3
SPDE1
SPDE2
SPDE3

Fig. S3.
